# Ethanol Disrupts Reactivated Contextual Conditioned Fear Memory: Behavioral and Histological Perspectives

**Published:** 2011-12-22

**Authors:** Jafar Alijan-pour, Kataneh Abrari, Taghi Lashkar bluki, Mohammad Taghi Ghorbanian, Iran Goudarzi, Mahmoud Elahdadi Salmani

**Affiliations:** School of Biology, Damghan University, Damghan, Iran

**Keywords:** Hippocampus, Reconsolidation, Conditioning, Ethanol

## Abstract

**Objective:**

This research study is an attempt to examine whether the administration of ethanol after memory reactivation would modulate subsequent expression of memory in rats. Additionally, we examined whether this administration alters the density of Cornu Ammonis (CA)1 and CA3 pyramidal and dentate gyrus (DG) granule cells.

**Materials and Methods:**

In this experimental study, adult male Wistar rats (200-300 g) were trained in a fear conditioning system using two 1 second, 0.6 mA shocks with an interval of 180 seconds. Twenty four hours later rats were returned to the chamber for 120 seconds. Immediately after reactivation they were injected with ethanol (0.5, 1, 1.5 mg/ kg) or saline. 1, 7 and 14 days after reactivation, rats were returned to the context for 5 minutes. Seconds of freezing (absence of all movement except respiration) were scored. In the second experiment (described in the previous paragraph), after test 1, animals were anesthetized with sodium pentobarbital and perfused transcardially with phosphate buffer (10 minutes) and 4% paraformaldehyde (15 minutes). The brains were postfixed in phosphate-buffered 4% paraformaldehyde (24 hours) and 30% sucrose. 10-µm sections were stained with cresyl violet.

Data were analyzed by 1-and 2-way ANOVA for repeated measurements by means of SPSS 16.0. Tukey's post hoc test was performed to determine the source of detected significant differences. P <0 .05 were considered significant. Data are presented as mean ± SEM.

**Results:**

Findings from the first experiment indicated that ethanol at a dose of 1.5 mg/kg significantly impaired recall of memory only in the first test. The density of CA1 and CA3 pyramidal and DG granule cells in the ethanol group was decreased (p< 0.01) compared with control group respectively 43.7%, 35.8%, and 37.8.

**Conclusion:**

The data demonstrate that ethanol exposure impairs post retrieval processes. Moreover, ethanol decreases the density of CA1, CA3 and DG cells. Presumably it would be a correlation between our behavioral and histological results.

## introduction

In experimental subjects, exposure to a conditioned stimulus (CS, such as a context) without the unconditioned stimulus (US, such as footshock) may initiate two potentially dissociable but opposite processes: extinction andreconsolidation. During reconsolidation the original memory is thought to update or integrate new information into longterm memories. Conversely,the extinction process tends to weaken the original memory (1-4).

Ethanol (alcohol) is a short chain lipid soluble compound whose initial mechanism of action was thought to affect the whole brain via a “lipid membrane disordering” effect. The rationale behind this mechanism results from the significant correlation between a number of alcohols and their partition coefficient between oil and water. However, it is now clear that ethanol alters brain neurobiology only in specific brain regions (5, 6). Over the last several years, a variety of projects have demonstrated that hippocampal neurophysiology and function is altered by ethanol (5, 7-8).

It has long been recognized that ethanol can have profound effects on learning and memory. Some reports indicate that post-training administration of ethanol dose-dependently decreases avoidance while others indicate that even very high doses of ethanol (4.5 g/kg) administered immediately after training improve retention. Still other research has reported no effects of post-training ethanol on retention of two types of avoidance tasks in rats (9, 10). Other reports have shown that immediate post-training injection of moderate ethanol doses in mice has little effect on context and cued fear conditioning (11, 12). However, conflicting results are reported in animal studies. Administration of ethanol post-training can either enhance or impair learning. Thus, the effects of post-training ethanol on a variety of tasks are quite complex. By contrast, little is known about the effect of ethanol on consolidated memories. Only one report appears to have been published. It shows that rats receiving ethanol with reactivation exhibited longer freezing than those given ethanol without reactivation, suggesting that ethanol does not inhibit the memory decline (eg, extinction), but facilitates fear memory (9, 13).

The effects of ethanol however, depend on several factors, including when ethanol is administered relative to training, the dose, and the type of task involved. Recent reviews suggest that ethanol may have particularly detrimental effects on hippocampus- dependent forms of memory. For example, acute pretraining ethanol administration to rodents compromises trace fear conditioning, contextual fear conditioning and spatial navigation, all considered to be hippocampally-mediated tasks, in a dose-dependent manner (14-16).

Using a microscopic approach in animal model systems, alcohol-induced morphological changes in the brain have been shown to be associated with significant cell loss in various neuronal populations including pyramidal cells in the hippocampus. Neurodevelopmental studies of the teratogenic actions of ethanol indicate that ethanol can retard cell proliferation and increase cell death particularly through apoptosis (17-19). Given the fact that ethanol readily crosses the blood-brain barrier and produces selective neurophysiological effects in the hippocampus, it seemed reasonable to investigate whether acute ethanol administration, after retrieval, selectively altered hippocampal-dependent contextual fear conditioning memory via altering the population of cells in this organ.through apoptosis (17-19). Given the fact that ethanol readily crosses the blood-brain barrier and produces selective neurophysiological effects in the hippocampus, it seemed reasonable to investigate whether acute ethanol administration, after retrieval, selectively altered hippocampal-dependent contextual fear conditioning memory via altering the population of cells in this organ.

## Materials and Methods

### Animals

Adult male Wistar rats (200-300 g) were used in this expremental study. Animals were housed five rats to a cage and maintained on a 12-hour light/dark cycle. Food and water were provided ad libitum. Behavioral tasks were performed during the light phase of the cycle. All procedures were conducted in agreement with the National Institutes of Health Guide for care and use of laboratory animals.

### Contextual Fear Conditioning Apparatus

An automated rodent fear conditioning system (Germany) was used to study contextual fear conditioning of each rat. Contextual fear conditioning took place in a conditioning box. The walls and the ceiling of the box were constructed of clear Plexiglass. The box was in an isolation cubicle (45 cm × 45 cm × 47 cm) containing a loud speaker and light bulb providing dim illumination. The floor of the box was made of 28 stainless steel rods (6 mm in diameter, 12 mm apart) through which foot shocks could be delivered from a constant current source. The box was enclosed in a sound attenuating chamber. The chamber was illuminated by a single house light, and was cleaned before and after utilization. A software program was used to control the test in the box, and to collect, display and store all experimental data for “off-line” analysis.

### Behavioral training and testing procedures Habituation

The day before the start of conditioning the rats were brought to the experimental room and placed individually in chamber A for 5 min and then returned to their home cages. Chamber A had some toys for the rats. Training

The conditioning session consisted of placing the rats in chamber B and delivering a footshock (CS) 180 seconds later. Both chambers A and B were identical in size and are the same, but chamber B had no toys. Two 1second moderate shocks of 0.6 mA with an interval of 180 seconds were administered. Rats were left in the conditioning box for 90seconds after termination of the procedure and returned to their home cage.

### Memory reactivation

On day 2, rats were placed in the same conditioning box for 120 seconds without receiving any shock. Immediately after memory reactivation, rats received one of the treatments mentioned below.

### Testing

One (test 1), 7 (test 2) and 14 (test 3) days after memory reactivation, ratswere returned to the box for 5 min. Memory was assessed and expressed asthe percentage of time that rats spent frozen. Such behavior is commonlyused as an index of fear in rats. Freezing was defined as the absence of allvisible movement expect respiration. The reactivation session and contextual testing were video recorded and automatically measured to score for freezing (20, 21).

### Experiment 1

This experiment examined the effects of the administration of various doses of ethanol following memory reactivation on post-retrieval processes in rats trained under moderate shock intensities.

#### Methods

Rats were randomly divided into 4 groups (n = 8-10 in each group) and trained according to the procedures described. Immediately following memory reactivation, the animals received saline or ethanol (0.5, 1 or 1.5 mg/kg). One day after memory reactivation, all animals were re-exposed for a 5minutes period to the training context and the time spent in a frozen position was recorded.

**Fig 1:**
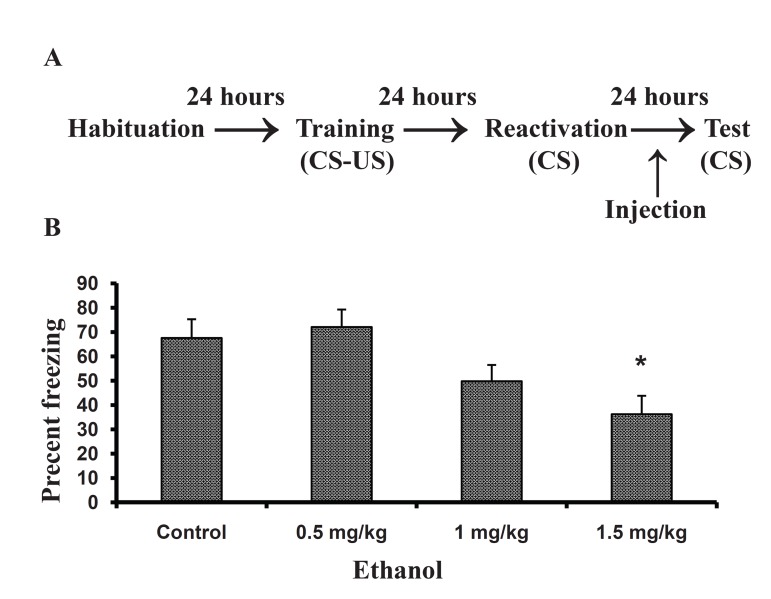
Effect of ethanol administration following memory reactivation of a contextual fear memory. A. Behavioral procedure of the experiment. Rats were trained, different doses of ethanol were injected immediately following memory reactivation (120 s) which was done 24 hours after training. The control group received saline, B. A retention test was done one day after memory reactivation. Data are expressed as means ± SEM of percent of time spent freezing during a 5 minutes retention test.* P < 0.05 compared with the control group.

Figure 1 shows the effects of treatment following memory reactivation on retention performance as assessed by the time spent frozen during a 5 min retention test one day after memory reactivation. One way ANOVA of the freezing data showed a significant effect of ethanol (F 3, 38 = 6.028; p = 0.002). Post-hoc comparison indicated that there is a significant difference between the saline group and the ethanol group receiving a dose of 1.5 mg/ kg (p < 0.02), but not 0.5 or 1 mg/kg.

### Experiment 2

In experiment 1, we found that ethanol at a dose of 1.5 mg/kg temporarily impairs subsequent retrieval in rats. The aim of experiment 2 was to examine maintenance of this ethanol effect.

#### Methods

Rats were randomly divided into 4 groups (n = 8-10 in each group) and trained according to the procedures already described. Seven (test 2) and 14 (test 3) days after memory reactivation rats were returned to chamber B for the context test.

#### Results

**Fig 2:**
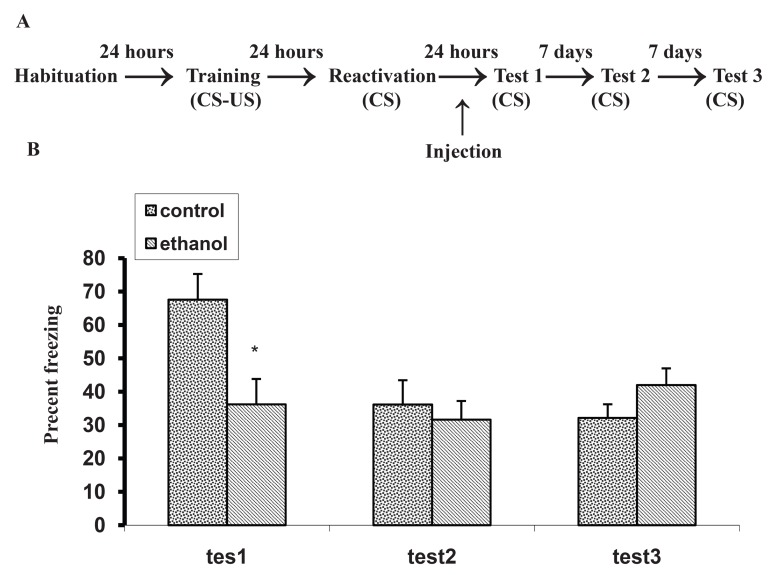
Effect of ethanol administration following memory reactivation on a contextual fear memory. A. Behavioral procedure of the experiment. Rats were trained. Different doses of ethanol were injected immediately following memory reactivation (120 s) which was done 24 hours after training. B. The control group received saline. Retention tests were done 1, 7 and 14 days after memory reactivation. Data are expressed as means ± SEM of percent of time spent freezing during a 5 minutes retention test. P < 0.01 compared with the control group.

Figure 2 shows the effects of treatment following memory reactivation on retention performance as assessed by the time spent frozen during a 5 minutes retention test, 1, 7 and 14 days after memory reactivation. A two way ANOVA with repeated measures analysis of the freezing response data revealed significant effects of ethanol ([F(2, 60)= 3.49; p = 0.04], and a significant effect of days [F(2, 60) = 4.34; P = 0.01], indicating a decay of freezing from the 1st test to 3rd test. There was no significant interaction between the effects of ethanol and time in days [F(4, 60) =2.32; p = 0.06]. This difference was only seen in the first, but not the second or third tests. These results may indicate that ethanol temporarily impairs post retrieval processes in rats.

### Experiment 3

In experiments 1 and 2 we found that administration of ethanol after memory reactivation impairs subsequent recall. If it selectively impairs retrieval of reactivated memories, no amnesic effects of these drugs would be observed in the absence of memory reactivation.

#### Methods

Rats were randomly divided into 2 groups (n =8-10 in each group) and trained according to the procedures described previously. Twenty-four hours later, they received saline or ethanol (1.5 mg/ kg) in their home cage (no memory reactivation) and their freezing responses were tested 24 hours later as indicated above.

#### Results

**Fig 3:**
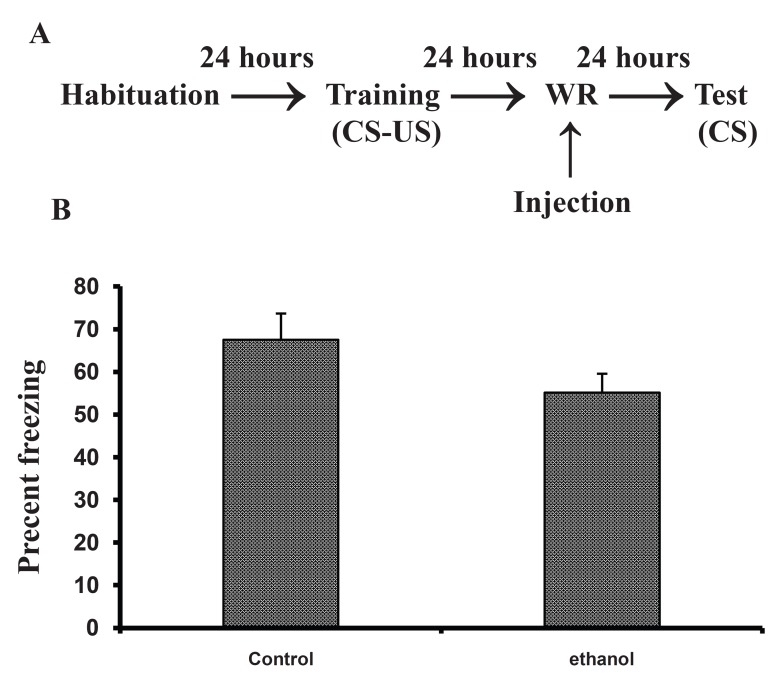
Effect of ethanol (1.5 mg/kg) in the absence of memory reactivation of a contextual fear memory. A. The behavioral procedure of the experiment. Rats were trained . Rats received ethanol (1.5 mg/kg) in their home cages 24 hours after training (no memory reactivation). A retention test was done 24 hours later. Data B are expressed as in figure 1.

Student's t test indicated that there was no significant difference between ethanol (t = 0.11, p = 0.08) (Fig 3) and saline. These results indicated that memory reactivation must occur for ethanol to alter post-retrieval memory processes.

### Experiment 4

In experiment 1, we found that memory deficit following ethanol administration is temporary. This could be attributable to a facilitated extinction process rather than impairment of memory reconsolidation. Since extinction of memory can be reversed by subsequent exposure to the reminder shock, this experiment examined whether the memory would recover from ethanol amnesia after a subsequent exposure to a weak and single footshock (0.4 mA, 1 second) in the training context as a reminder shock.

#### Methods

Rats were randomly divided into 4 groups (n = 18-20 in each group) and trained according to the procedures described previously. Memory reactivation (60 seconds) occurred 24 hours later. Immediately after memory reactivation, the rats received saline or ethanol (1.5 mg/kg). Four hours after memory reactivation, animals were relocated into the context for 1 minute and half of the animals in each group received a single 1 second footshock of 0.4 mA as a reminder shock (RS) and half of animals did not (no RS).

#### Results

**Fig 4:**
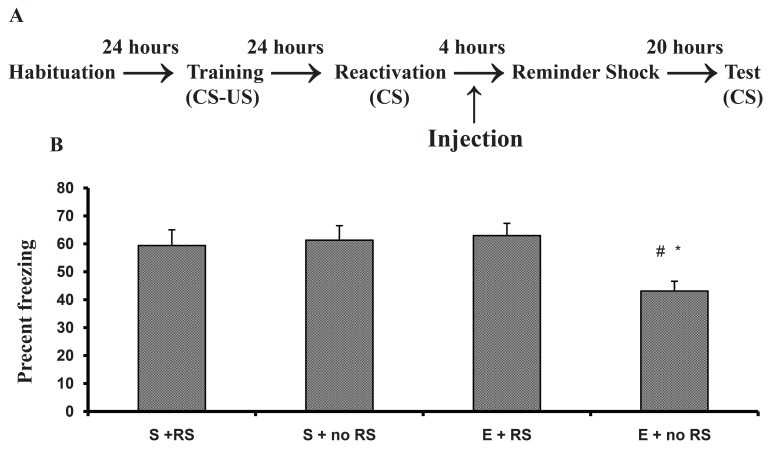
Effect of a reminder shock (RS) on ethanol induced amnesia. A. The behavioral procedure of the experiment. Memory reactivation (120 s) occurred 24 hours after training. Immediately after memory reactivation, rats received Saline (S) or ethanol (1.5 mg/kg). Four hours after memory reactivation, animals were relocated into the context and half of the animals in each group received a single RS (1 s, 0.4 mA) and half of the animals did not (no RS). # P < 0.01 compared with the corresponding control group. * P < 0.02 compared with ethanol + RS and ethanol + noRS group.

One way ANOVA analysis of the freezing data indicated significant differences between saline/ noRS and ethanol/noRS (p < 0.01), ethanol/RS and ethanol/noRS (p< 0.02). There was no significant difference between saline with or without RS and ethanol/RS (Fig 4). These results indicated that a weak reminder shock reverses the ethanol effect.

### Experiment 5 Histological Methods

As in experiment 1 the animals were trained. Then after the first test rats were anesthetized intrapritoneally with a mixture of ketamine (100 mg/ kg) and xylazine (4 mg/kg) and perfused intracardially with 0.1 M phosphate buffer for 10 minutes followed by phosphate-buffered 4% paraformaldehyde for 15 minutes.

### Infiltration and embedding

The brains were removed and the right hippocampus was dehydrated through a graded series of alcohols (50%, 60%, 70%, 80% for 1 hour each, 90% and 96% for 1.5 hours each and 100% twice for 1.5 hours) prior to infiltration. After dehydration, clearing and impregnation the hippocampal blocks were then embedded in disposable tissue molds (22-24).

### Staining

Five coronal sections (10 µm) from each animal were cut at the level of the dorsal hippocampus and stained using cresyl violet. The staining solution contained 0.5g cresyl violet dissolved in 100ml distilled water. The mounted sections were placed in the staining solution for 20-30 minutes at room temperature, differentiated in 0.25% acetic acid until most of the stain had been removed (4-8 seconds) and then briefly passed through absolute alcohol into xylene and checked microscopically. If it was necessary differentiation was repeated. Sections were then cleared with xylene and the coverslip bonded with Entellan. The number of pyramidal cells in a 130-µm segment of each of the hippocampal CA1 and CA3 fields and granule cells in the dentate gyrus were counted using light microscopy at × 400 magnification (22-24).

### Definitions of hippocampal cell layers

The principal neurons in the different subdivisions of the hippocampus were clearly differentiated from each other. Neurons were counted based on identification of a clear and distinct nuclear membrane, and counting was restricted to the right hippocampal formation. The cell bodies of CA3 are large, elongated and tightly packed in a layer four to five cells deep.

The cell bodies and nuclei of the pyramidal cells of CA1 are smaller than those of CA3.The granular layer of the DG contains the smallest and most densely packed cell bodies in the hippocampus. The cell bodies are packed 8-15 cells deep and have well defined borders. In addition the layer is not in immediate contact with other densely packed layers. The number of surviving neurons from three to four sections per animal at the dorsal hippocampal level was counted by a blinded observer using light microscopy. Only whole neurons with a visible nucleus were counted. The data were expressed as surviving cell numbers per mm in each region of the hippocampus (22-24).

## Results

### Number of CA1 pyramidal cells

**Fig 5:**
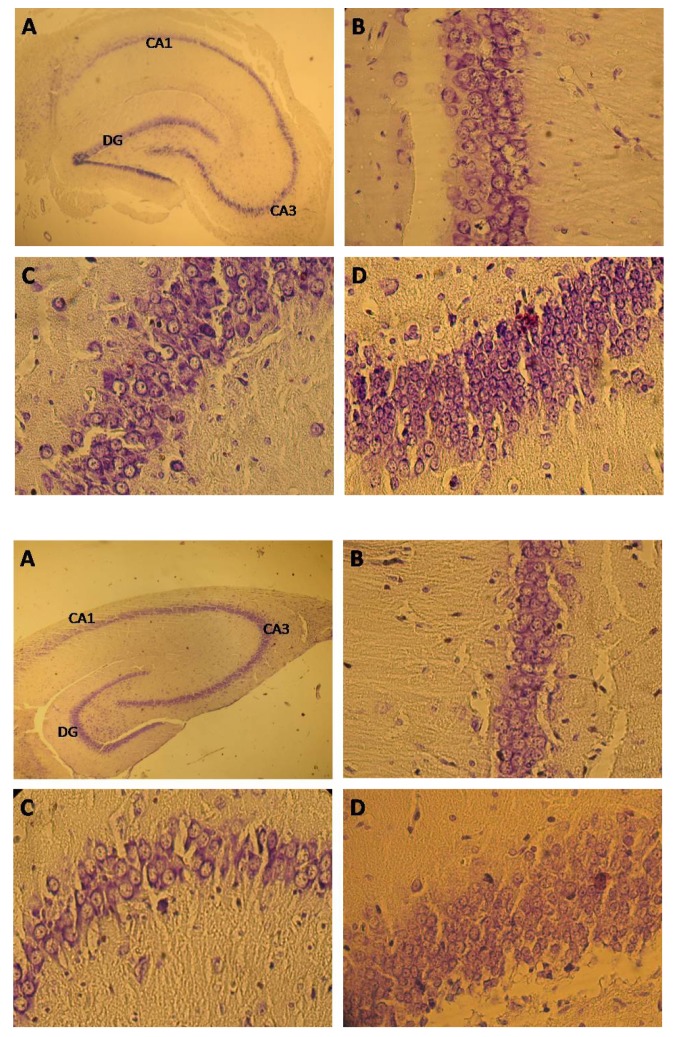
Nissl staining of hippocampal cells. Upper 4 photos (1) are for the control group. Lower 4 photos (2) are for the ethanol group. A: Whole section of hippocampal formation. B:CA1, C: CA3, D:DG

**Fig 6:**
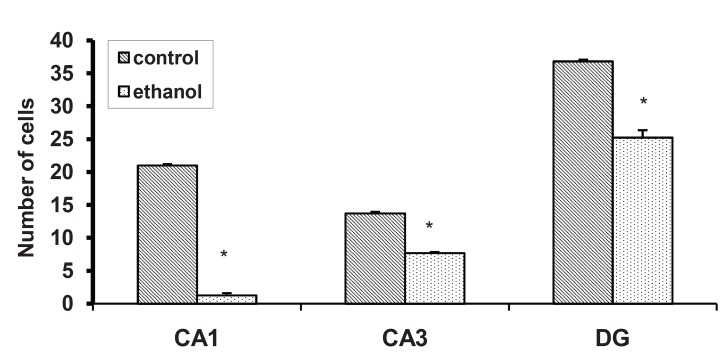
The number of pyramidal cells in a 130-µm2 segment of different regions of the hippocampus. The number of CA1 and CA3 pyramidal cells and DG cells in the ethanol treated group showed a significant decrease compared with the control group (p < 0.0001).

Figure 5 represents the photographs of coronal sections containing the hippocampal CA1 region. The number of pyramidal cells in the ethanol treated group was significantly less (35.84%) than in in the control group (p < 0.0001) (Fig 6). The average number of neurons in six sections of each of the ethanol group animals is shown in table 1.

### CA3 pyramidal cells

The number of pyramidal cells in a 130-µm2 segment of the hippocampal CA3 field was significantly different among two groups (p < 0.0001) (Figs. 5, 6). The average number of neurons showed a 43.7% decrease in the ethanol treated group compared with the control group. The average number of neurons in six sections of each of the animals is shown in table 1.

### DG granule cells

The total number of DG neurons was significantly different among groups (p < 0.0001) (Figs. 5, 6). The average number of DG neurons showed a 37.83% decrease in the ethanol treated group compared with the control group (Table 1).

### Blood alcohol concentrations

Blood alcohol concentrations (BAC) were not determined in the present experiment, with the reasoning that any stress induced by the blood sampling procedure after fear conditioning may unduly influence learning, and thus confound results. However, analysis of the pharmacokinetics of i.p. alcohol in the rat shows that BAC rapidly rises and peaks at about 5 min post-injection.

**Table 1:** The average number of neurons in six sections of each animal (n=10 animals in each group). Data are expressed as means ± SEM


	CA1	CA3	DG
Control	21 ± 0.57	13.7 ± 0.75	36.85 ± 0.68
Ethanol	13.28 ± 0.95	7.71 ± 0.48	25.28 ± 3


## Discussion

The main purpose of the present study was to investigate the effects of systemic injection of ethanol following memory reactivation on subsequent expression of fear memory in rats. We have demonstrated that ethanol reduces reactivated contextual fear memory. Rats receiving ethanol after reactivation demonstrated shorter durations of freezing during the contextual test.

The results indicate that the administration of ethanol after memory reactivation temporarily impaired subsequent retrieval of a contextual fear memory in rats. This ethanol effect is temporary because it lasts only for one week (test 1) and not for 7 days (test 2) or 14 days (test 3).

This impairment is only seen after memory reactivation and not in the absence of memory reactivation, indicating that adequate memory reactivation must occur for ethanol to alter post reactivation memory processes. Injection of ethanol without reactivation 24 hours after conditioning had no effect. Taken together, we can say that ethanol affects the retrieval induced process and reduces reactivated contextual fear memory.

Previous studies suggest that 2minutes reactivation changed consolidated memory into a labile state and then induced the reconsolidation process, which required de novo protein synthesis (25). Since the effect of ethanol is limited to shorter reactivation, which induces the reconsolidation process, we consider that ethanol enhances fear memory through memory reconsolidation. On the other hand, previous studies have shown that amnesia due to an extinction trial can be defeated by a weak reminder shock (26). In experiment 4, the single and brief re-exposure (120 seconds) to the associated context did not provoke a demonstrable amnesia in control animals (Fig 4), supporting the notion that this time of exposure is not enough to initiate extinction. However, a weak reminder shock reverses the ethanol-induced amnesia effect.

In the present study, post-reactivation ethanol induced a retention deficit of about 30.75% in the first test (possibly replace with after 1 day). In the work of Davis and Rosen zweig, anisomycininduced impairment of about 35% in memory recall (27). In both cases, recovery of memory impairment occurred over time. In contrast, in a study by Debiec et al. the magnitude of memory recall impairment was 80%, which did not recover over time (28). Thus, the amount of deficit in the first retention test after treatment might be one important factor in determining the possibility for recovery from amnesia: less impairment suggests a greater probability of recovery.

A recent series of studies in models of working memory, suggested two important points: first, that the effects of alcohol on memory are dosedependent, and second, that learning in different paradigms, possibly involving divergent neuronal populations, may be differentially affected by alcohol. In fact, it has been recently shown that hippocampus dependent context learning is blocked by a moderate dose of alcohol (1.0-1.5 g/kg), but not by 0.5 g/kg alcohol, whereas hippocampus-independent cued conditioning remains insensitive to these doses of alcohol. A larger dose of alcohol produced general suppression of activity and led to an attenuation of both hippocampal-dependent and independent fear conditioning, suggesting a more general intoxicating effect (29- 31). In our experiments the effective dose of ethanol was 1.5 g/kg in common with some other recent reports. What are the cellular mechanisms underlying the degradation of memory? The hippocampus plays an important role in reconsolidation of contextual fear memory. There is a report that ethanol preferentially affects the hippocampus in contextual fear conditioning. Hippocampal place cells have been proposed as the cellular map underlying spatial information processing in the hippocampus. Given that acute ethanol administration impairs spatial memory, it seems reasonable to predict that similar doses of ethanol should alter the spatial specificity of hippocampal place cells. It was first reported that a 1.0 g/kg ethanol injection decreased the number of place “units” recorded from awake freely behaving rabbits. A more systematic study using rats investigated the effect of a higher dose of ethanol, 2.0 g/kg, on the spatial specificity of place cells (32-34).The exact cellular mechanism by which acute ethanol administration impairs spatial memory is unknown. In previous experimental studies, it has been shown that short-term administration of ethanol results in many changes, such as a decrease in different types of cells in the hippocampus. For example, a 5-g/kg dose of ethanol decreased adult neural progenitor cell proliferation in the adolescent rat dentate gyrus, forebrain regions and sub ventricular zone by 40%. It has also been reported that an acute dose of ethanol decreased the number of BrdU+ cells in the adult hippocampus 5 hours after administration. Nevertheless the prior evidence suggests that acute alcohol consumption may first initiate programmed cell death, an effect that is then followed by passive non-programmed degeneration(35-37).

Earlier investigators evaluated only the numbers of specific cell types such as BrdU+ or neural progenitor cells, whereas researchers in the present study estimated the total number of cells. It is proposed here that ethanol may cause a decrease in the total number of cells, but we do not know its effect on numbers of specific cell groups.

In contrast to these findings, other researchers have found that an acute dose of ethanol (3 g/kg) did not significantly change the total number of neurons in the right hippocampus of the rat (37). However such reports are very rare. In our study, neurons were counted in a defined area of each of the CA1 and CA3 and dentate gyrus regions of the hippocampus. An average value was calculated based on the analysis of several brain sections or several regions in a brain section of the individual animals that were examined. This histological approach provides an assessment of the number of neurons in a defined area of a particular brain region and allows the determination of an ethanol treatment effect on hippocampal pyramidal cell and dentate gyrus granule cell density. In our data all three areas of the hippocampus were sensitive, but most the sensitive was the CA3 region.

Ethanol is a complex neurotoxin; the precise mechanisms by which it causes neuropathological changes are not clearly defined. More recently, oxidative stress mediated apoptosis has received much attention in the search for underlying mechanisms. Alcohol promotes the generation of reactive oxygen species (ROS) and/or interferes with the body's normal defense mechanisms against these compounds through numerous processes, particularly in the liver. For example, alcohol breakdown in the liver results in the formation of molecules whose further metabolism in the cell leads to ROS production. Alcohol also stimulates the activity of enzymes called cytochrome P450s, which contribute to ROS production. Further, alcohol can alter the levels of certain metals in the body, thereby facilitating ROS production. Finally, alcohol reduces the levels of agents that can eliminate ROS (i.e., antioxidants). The resulting state of the cell, known as oxidative stress, can lead to cell injury. Reactive oxygen species (ROS) are small, highly reactive, oxygen-containing molecules that are naturally generated in small amounts during the body's metabolic reactions and can react with and damage complex cellular molecules such as fats, proteins, or DNA. Alcohol promotes the generation of ROS and/or interferes with the body's normal defense mechanisms against these compounds through numerous processes, particularly in the liver. For example, alcohol breakdown in the liver results in the formation of molecules whose further metabolism in the cell leads to ROS production. Alcohol also stimulates the activity of enzymes called cytochrome P450s, which contribute to ROS production. Further, alcohol can alter the levels of certain metals in the body, thereby facilitating ROS production. Finally, alcohol reduces the levels of agents that can eliminate ROS (i.e., antioxidants). The resulting state of the cell, known as oxidative stress, can lead to cell injury (38). Ethanol can cross cell membranes readily, including the blood-brain barrier. The hippocampus is a brain area particularly vulnerable to ethanol-induced oxidative stress (39). Additional studies will be needed to determine the detailed mechanisms of ethanol-induced memory deficit and it's relation to oxidative stress. Other additional studies are required to further clarify how alcohol produces oxidative stress in various tissues. For example, more detailed information is needed on the mechanisms involved in some of the major proposed pathways (e.g., how alcoholderived NADH leads to ROS production either directly or during the passage of NADH-derived electrons through the mitochondrial respiratory chain). Other mechanisms remain highly controversial, such as the role of CYP2E1 or of various cytokines in alcohol-induced oxidative stress (40). Additional analyses need to determine the role of alcohol metabolism and its byproducts (e.g., acetaldehyde) in the production of ROS. Finally, it still is unclear how alcohol-induced oxidative stress is produced in tissues where only limited alcohol metabolism occurs.

## Conclusion

Our data demonstrate that ethanol exposure impairs postretrieval processes. Our results indicate that ethanol administration after memory reactivation produced a transient deficit in the subsequent expression of memory. Memory retrieval triggers memory reconsolidation and extinction. We explored the possibility that ethanol affects the reconsolidation process.

In our study neurons were counted in a defined area of each of the CA1 and CA3 and dentate gyrus regions of the hippocampus. We observed that ethanol treatment effected the total number of neurons in the hippocampus, but the area most sensitive to neuron depletion was the CA3 region. Presumably it would be a correlation between our behavioral and histological results.
